# Dorsal Lateral Geniculate Substructure in the Long–Evans Rat: A Cholera Toxin B Subunit Study

**DOI:** 10.3389/fnana.2012.00040

**Published:** 2012-09-25

**Authors:** Claire B. Discenza, Pamela Reinagel

**Affiliations:** ^1^Department of Neuroscience, School of Medicine, University of CaliforniaSan Diego, CA, USA; ^2^Section of Neurobiology, Division of Biological Sciences, University of CaliforniaSan Diego, CA, USA

**Keywords:** retinal projection, thalamus, visual, pigmented rat, lamination, segregation, binocularity

## Abstract

The pigmented rat is an increasingly important model in visual neuroscience research, yet the lamination of retinal projections in the dLGN has not been examined in sufficient detail. From previous studies it was known that most of the rat dLGN receives monocular input from the contralateral eye, with a small island receiving predominantly ipsilateral projections. Here we revisit the question using cholera toxin B subunit, a tracer that efficiently fills retinal terminals after intra-ocular injection. We imaged retinal termini throughout the dLGN at 0.5 μm resolution and traced areas of ipsilateral and contralateral terminals to obtain a high resolution 3D reconstruction of the projection pattern. Retinal termini in the dLGN are well segregated by eye of origin, as expected. We find, however, that the ipsilateral projections form multiple discrete projection zones in three dimensions, not the single island previously described. It remains to be determined whether these subdomains represent distinct functional sublaminae, as is the case in other mammals.

## Introduction

In mammals, the retinal ganglion cells (RGC) that contribute to cortical vision send their projections to the dorsal lateral geniculate nucleus (LGN) of the thalamus (dLGN). Ipsilateral and contralateral projections remain segregated at the level of the dLGN (Matteau et al., 2003). In many species, the projections of distinct ganglion cell types further segregate into discrete dLGN laminae, each containing a retinotopic map of visual space (Bishop et al., 1962; Laties and Sprague, 1966; Garey and Powell, 1968; Kinston et al., 1969; Sanderson, 1971a,b; Jones, 2007, for review). Although species differ in their retinal ganglion cell classes and the in the details of their dLGN lamination, the segregation of information into anatomically separate parallel processing streams is a conserved organizing principle of the dLGN (Cleland et al., 1971a,b; Kaas et al., 1972; So et al., 1990; Roy et al., 2009; see Sherman and Guillery, 2000 for review).

For example, the dLGN of the macaque monkey (*Macaca mulatta*) contains six layers, each receiving inputs from a different subset of RGC (parasol or midget; ON or OFF; ipsilateral or contralateral) (Malpeli and Baker, 1975; Schiller and Malpeli, 1978; Connolly and Van Essen, 1984; Shapley and Perry, 1986; Szmajda et al., 2006; Murray et al., 2008). In the cat (*Felis catus*), six layers have been distinguished in the dLGN, receiving inputs from different subsets of RGC types (X, Y, or W) and segregated by eye of origin (Guillery, 1970; Sherman and Spear, 1982; Shapley and Perry, 1986). The ferret (*Mustela putorius furo*) is similar to cat with further sublamination of ON and OFF types (Stryker and Zahs, 1983). In the California ground squirrel (*Spermophilus beecheyi*), a diurnal rodent, the retinal projections form three layers with alternating eye of origin in the dLGN; six sublaminae have been distinguished (Roe et al., 1989).

It remains unclear, however, whether the dLGN is as highly organized in nocturnal rodents such as the rat and mouse, which lack obvious lamination in the Nissl preparation (see Jones, 2007, for review). Nevertheless, these nuclei are not homogenous. In the mouse (*Mus musculus*), distinct functional classes of RGCs have been found to project to distinct layers in the dLGN (Huberman et al., 2008, 2009). In the rat (*Rattus norvegicus*), the nucleus has been subdivided into two general regions by anatomy and physiology: an outer lateral “shell” and an inner medial “core” (see Reese, 1988 for review). These two regions differ in that they receive projections from differing populations of morphological ganglion cell types (Bunt et al., 1974; Hickey and Spear, 1976; Fukuda, 1977; Brauer et al., 1979) and contain distinct morphological classes of relay cells and termini (Lund and Cunningham, 1972; Bartlett and Smith, 1999). In addition, the outer “shell” receives input from the optic tectum (Reese, 1984), and the inner “core” contains a smaller internal region which receives termini emanating from the ipsilateral eye (Reese and Cowey, 1983). It has been shown that these segregated zones contain their own retinotopic map of visual space, although only the contralateral outer “shell” region is known to contain a complete map (Montero et al., 1968; Reese and Jeffrey, 1983; Reese, 1988). Most studies report segregation of inputs by eye of origin in the dLGN of pigmented rats (Reese, 1988; Guido, 2006; but see Hayhow et al., 1962; Grieve, 2005).

In the current study, we used Cholera Toxin B subunit (CTB), which is not only a retrograde tracer but also an efficient anterograde tracer (Angelucci et al., 1996). We injected CTB intra-ocularly, which has been shown to efficiently fill retinal ganglion cell termini in their subcortical targets (Reiner et al., 1996; Matteau et al., 2003). We traced the retinal termini to determine the volume of the dLGN relative to two other retinorecipient structures: the optic tectum and the ventral lateral geniculate (vLGN). We reconstructed a three-dimensional model of the ipsilateral and contralateral projections to the dLGN to determine if there is more than one discrete projection zone for either eye. Finally, we determined the accuracy of segregation into eye-specific domains within the dLGN.

## Materials and Methods

### Subjects

**Figure A1:**
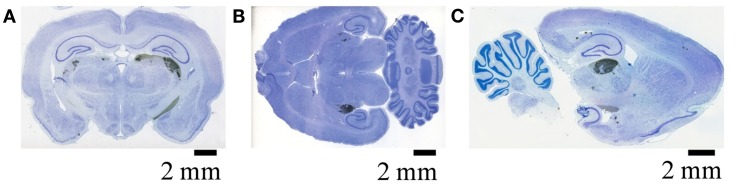
**Retinal termini in the dLGN as visualized by DAB**. Retinal termini labeled by monocular intra-ocular injection of Cholera toxin B subunit, processed by DAB reaction, and imaged by the Aperio slide scanner. Filled retinal termini are visualized as DAB reaction product (black), cell bodies by Giemsa counterstain (blue) (see Materials and Methods of main manuscript for details). **(A)** Coronal section showing termini in the ipsilateral (left) and contralateral (right) dLGN. **(B)** Horizontal section from a different subject, showing the dLGN ipsilateral (top) and contralateral (bottom) to injected eye. **(C)** Saggital section from a third subject, passing through the dLGN contralateral to the injected eye.

**Figure A2:**
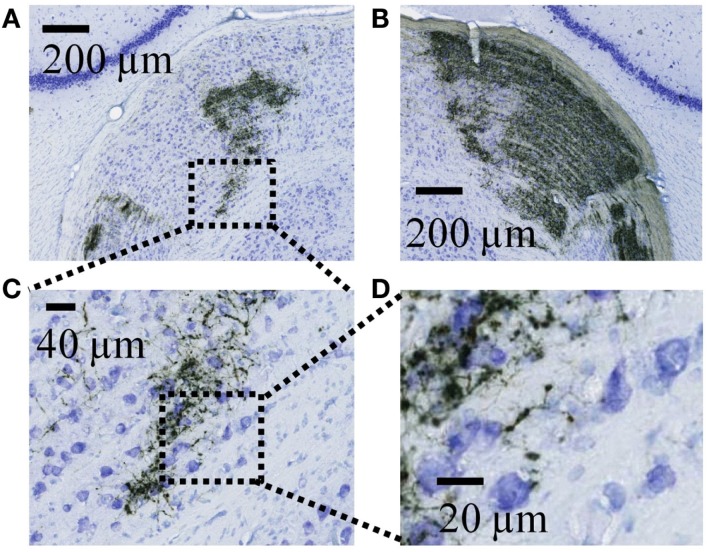
**High resolution views of retinal termini in the dLGN as visualized by DAB**. **(A)** Expanded view of the ipsilateral dLGN from the image shown in Figure A1A. Dashed lines indicate the field expanded further in panel C. **(B)** Expanded view of the contralateral dLGN from the right side of the image in Figure A1A. **(C)** A further expanded view of the region indicated in panel A. **(D)** Expanded view of the region indicated in panel F, showing the detail at which individual retinal axons and termini were visualized during tracing.

**Figure A3:**
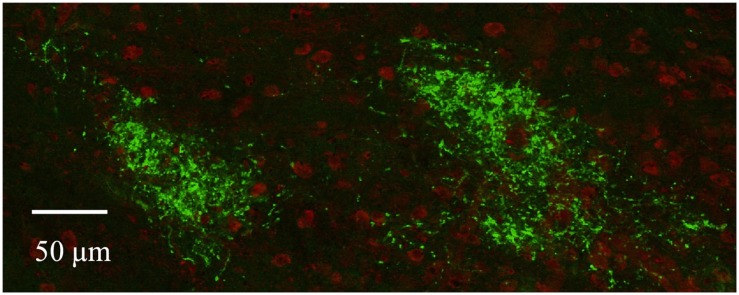
**Ipsilateral subdomains in the dLGN as visualized by confocal microscopy**. Retinal termini labeled by monocular injection of fluorescent CTB, imaged by confocal microscopy. Discrete spatially separated zones of retinal termini (green) are seen in the dLGN ipsilateral to the eye of injection in this and other sections. Cell bodies are stained red with NeuroTrace (Molecular Probes).

We examined retinal termini in subcortical targets in a total of 19 normal adult male Long–Evans rats (Harlan Laboratories, Inc.). Brains from subjects with monocular injections of unconjugated CTB were sectioned in either sagittal (*n* = 2), horizontal (*n* = 2), or coronal (*n* = 5) planes, processed by DAB, and imaged with the Aperio scanner (see below; Figures A1 and A2 in Appendix). Brains from subjects with monocular or binocular injections of fluorescently conjugated CTB were all sectioned in coronal plane. Most of these (*n* = 9) were prepared in thin sections for imaging with the Nanozoomer scanner (described below); one sample was prepared in thick sections and examined by confocal microscopy instead (Figure A3 in Appendix).

In all 19 specimens, we observed a high degree of segregation by eye of origin, and multiple spatially separated subregions of ipsilateral projections in the dLGN in at least some sections. Here we present quantitative analysis of these observations for the seven specimens that met two inclusion criteria: first, we had a complete series of undamaged sections spanning the entire dLGN and at least two sections on either side of it; and second, we observed complete staining of retinal termini throughout the entire dLGN sufficient to allow reliable tracing in every section. Rats 1–4 received binocular injections of fluorescently labeled CTB (see below) at 3–4 months of age (370–440 g). Subjects 5–7 received monocular injections of CTB at 6–7 months of age (490–670 g).

All subjects were maintained on a 12-h light/dark cycle with free access to food and water. All procedures were supervised and approved by the Institutional Care and Use Committee at the University of California, San Diego, USA.

### Intra-ocular injections

The B subunit of the Cholera Toxin complex (CTB) has been shown to be a highly sensitive anterograde tracer for RGCs (Angelucci et al., 1996; Reiner et al., 1996; Matteau et al., 2003), and therefore the preferred tracer for this study. Rats were first anesthetized with 2–5% isoflurane mixed with oxygen at a flow rate of 1 l/min., using an isoflurane vaporizer (Smiths Medical, Dublin, OH, USA) While maintained at the appropriate level of anesthesia, subjects were subcutaneously injected with buprenorphine (0.06 mg/kg rat weight). Subjects then received, via syringe, 5–6 2 μl injections of either unconjugated CTB in one eye, or fluorophor-conjugated CTB in both eyes.

The monocular injections were administered into the vitreous chamber of the left eye only, and comprised a 1% CTB solution (List Biological Laboratories, Inc., Campbell, CA, USA) mixed with 2% dimethyl sulfoxide (DMSO) diluted in sterile water. For binocular injections, rats were injected with two different fluorophor CTB conjugations, one in the vitreous chamber of each eye. A 1 mg/ml dilution of Alexa Fluor 488-conjugated CTB (Molecular Probes Inc., Eugene, OR, USA) in PBS was injected into the left eye, and a similar dilution of Alexa Fluor 594-conjugated CTB was injected into the right eye (Molecular Probes Inc., Eugene, OR, USA).

We waited 5–7 days post-injection before perfusion to allow for transport of tracer to the retinal termini (Wu et al., 1999). During this post-injection survival period subjects received twice-daily buprenorpnine injections for a minimum of 2–3 days, continuing as needed until sacrificed for perfusion and histology.

### Perfusion and histology

Five to seven days post-injection, all rats were euthanized with an overdose of isofluorane and perfused transcardially with 0.1 M phosphate buffered saline (PBS; pH 7.4) followed by 4% paraformaldehyde in PBS. After removal, brains were further fixed in 4% paraformaldehyde for at least 3 days, after which they were then soaked in a 30% sucrose PBS buffer solution for cryoprotection prior to slicing. Brains were sliced on a freezing microtome (Microm International GmbH, Waldorf, Germany); brains from non-conjugated monocularly injected rats were sliced at 30 μm in one of the three planes, and binocularly labeled brains were sliced at 25 μm coronally.

Fluorescent samples were sliced, separated into four series and mounted with Prolong Gold anti-fade reagent medium (Molecular Probes Inc., Eugene, OR, USA) on charged slides (Thermo Fisher Scientific Inc., Pittsburgh, PA, USA) and covered with a cover slip. After the initial round of imaging, slides were soaked to remove the cover slip, photo-bleached, and stained with NeuroTrace 500/525 nm green fluorescent Nissl stain (Molecular Probes Inc., Eugene, OR, USA) for other analyses described elsewhere (Discenza, 2011).

Non-fluorescent tissue samples were processed according to the method described by Angelucci et al. (1996) and Matteau et al. (2003). In summary, tissue was rinsed in phosphate buffered saline, and then incubated and rotated at 4°C overnight in a primary antibody solution of 0.1% Triton X-100, 5% normal rabbit serum, and a 1:1000–1:2000 dilution of biotinylated goat anti-rabbit CTB (List Biological Laboratories, Inc., Campbell, CA, USA cat #103B) in phosphate buffered saline. After rinsing again with phosphate buffered saline, tissue was then incubated, and slowly rotated for 1 h at room temperature in the secondary antibody solution consisting of a 1:1000 dilution of Vectastain biotinylated IgG (Vector Labs, Burlingame, CA, USA #PK-4005) with 0.3% Triton X-100 in phosphate buffered saline. Finally, after a third set of rinses, tissue was incubated in a tertiary antibody solution made using the Vectastain ABC kit ElitePK-6100 kit (Vector Labs, Burlingame, CA, USA). Tissue was incubated in a complexed avidin-biotin-peroxidase solution diluted to 1:1000 in phosphate buffered saline with 0.3% Triton X-100 and additional 2% NaCl. To visualize the CTB, the tissue was rinsed in buffer and soaked in a 1:3000 hydrogen peroxide phosphate buffer solution with 0.125 mg/ml Diaminobenzidine (DAB) for approximately 1 min, or until cells reacted. Tissue was rinsed, mounted on gel-coated slides (Thermo Fisher Scientific Inc., Pittsburgh, PA, USA), enhanced with 4% osmium, and coverslipped. One series from each brain was reacted with DAB alone, one series was counter-stained with Giemsa as well as DAB, and another series was Nissl-stained for other analyses described elsewhere (Discenza, 2011).

### Imaging

Fluorescent samples were imaged on the NanoZoomer 2.0 HT digital slide scanner (Hamamatsu Photonics, Japan). Slides were imaged at 20× resolution (0.46 μm^2^/pixel) using the fluorescent cube (DAPI/Fluorescein isothiocyanate/TexasRed). The non-fluorescent DAB/Giemsa series were scanned using Aperio Scanscope XT digital slide scanner (Aperio Technologies Inc., Vista, CA, USA; Burnham Institute, La Jolla, CA, USA) at 20× resolution (0.5 μm^2^/pixel), aligned using ImageJ software (Abramoff et al., 2004), and analyzed using custom software written in MATLAB (2008a–2010a, The MathWorks, Natick, MA, USA).

We confirmed a successful ocular injection by verifying uniform and complete staining of retinal termini throughout the optic tectum, as well as across the dLGN. This criterion is more stringent than inspecting staining in the retina, because it requires both complete filling of the retinal ganglion cell bodies and complete transport to retinal termini across the entire visual field.

### Tracing and 3D reconstructions

The dLGN termination zones were hand-traced over the high resolution digitized images of filled RGC termini, using the software Neurolucida (MBF Biosciences, Inc., Williston, VT, USA). The Rat Brain Stereotaxic Atlas (Paxinos and Watson, 1998) was used for initial identification of nuclei.

Outlines were traced delineating: (a) the entire dLGN, tracing the outer edge of both ipsilateral and contralateral termini, (b) ipsilateral subregions, or contiguous regions containing puncta from the ipsilateral RGC termini, (c) and the “holes” in the contralateral zones, or contiguous regions within the dLGN lacking contralateral retinal projections. Termination zones were traced while visualizing one fluorophor at a time. The aim was to encircle contiguous regions of retinal projections, and to separate these regions only when the distance between them was large compared to the termini density within the regions.

**Figure 1:**
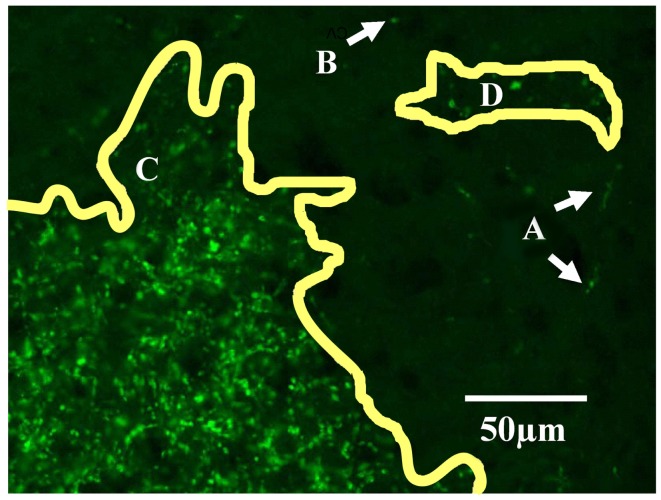
**Tracing criteria for termination zones**. The boundaries of retinal projection zones were manually traced from wide field (whole-brain), high resolution (0.46 μm/pixel) digitally scanned images, such as this field of view showing ipsilateral projections in part of the dLGN. At this resolution we can distinguish fibers of passage **(A)** from retinal termini; fibers of passage were ignored when outlining projection zones. Isolated single termini **(B)** were occasionally observed, and were ignored when outlining projection zones. Outlines were drawn around clusters of termini by smoothly connecting the outermost retinal termini within the cluster **(C,D)**. In the more densely populated projection zone shown here **(C)**, termini were overlapping to 10 μm apart. Clusters of termini that were far apart relative to the inter-terminal distances within the clusters were assigned to separate outlines **(C vs. D)**. Separate outlines could potentially be assigned to the same subregion in the 3-dimensional reconstruction (see Figure 2).

Projection zones were outlined manually according to defined tracing criteria (Figure 1). First, fibers of passage were observed but not included when defining the outline of a region. Fibers of passage typically fluoresced more faintly, and formed extended axonal shapes and not dense bright groups of puncta as did termini (Figure 1A). Secondly, areas of very low density were ignored, for example, areas containing fewer than one or two termini in 300 μm ^2^ (Figure 1B). Areas of high density, where puncta were either overlapping or up to 10 μm apart (Figure 1C), were traced, as were areas of low density, where puncta were 10–20 μm apart (Figure 1D). Outlines were drawn approximately 5 μm around the “outer boundary” of a termination zone, here defined as the outer termini of a zone where the next nearest neighboring puncta or zone is approximately 20 μm away.

We traced all sections throughout one specimen (Rat 1) and every fourth section throughout the remaining three fluorescent specimens (Rats 2–4). The outlines were aligned with Neurolucida, using ventricles, blood vessels, fiduciary pin marks, and dLGN outlines as landmarks. The aligned outlines were then stacked in Neurolucida to create a three-dimensional model of the dLGN. The same procedures and criteria were used to trace projection zones in the non-fluorescent, DAB-stained specimens from monocularly injected rats (Rats 5–7), but using custom Matlab software. Results did not differ in the two data sets.

### Assignment of termini to subdomains

Using the three-dimensional models, which can be rotated and examined from all angles, individual subdomains of retinal termination were distinguished by defined criteria in the four binocularly labeled specimens (Rats 1–4). Previous reports described only one compact ipsilateral termination zone in the rat dLGN, so our null hypothesis was that all ipsilateral projection outlines on each section through the dLGN were part of the same ipsilateral subdomain in three dimensions, despite appearing separated in the two dimensional plane. Therefore we were conservative in postulating separate subdomains, requiring that overlapping outlines were found in adjacent sections spanning at least 200 μm, and separation of at least 75 μm between it and its closest neighboring subdomain. Thus for specimen 1, in which every 25 μm section was traced, we required eight consecutive sections to contain an overlapping outlined termination zone, and at least three sections distance from the closest neighboring subdomain. For specimens 2–4, every fourth 25 μm section was traced, and we required three in a row to contain an overlapping region.

**Figure 2:**
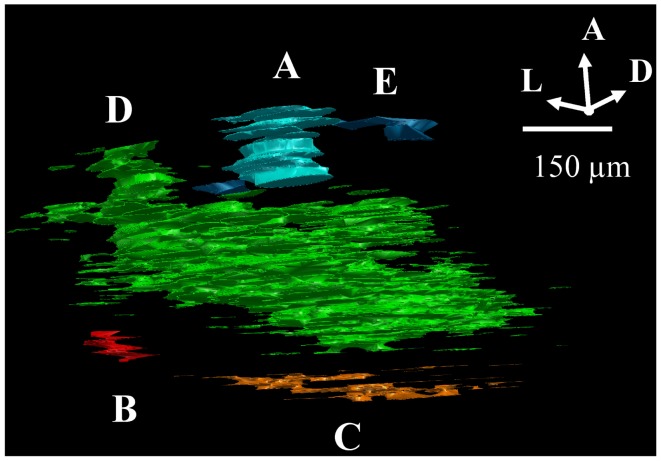
**Criteria for assigning 2D outlines to 3D subdomains**. **(A)** Example of a “dense subdomain,” defined as an anterior-posterior z-stack of ipsilateral outlined regions, each of which are densely populated by ipsilateral termini (Figure 1C), and whose outlines in neighboring sections overlap at least 10%. **(B)** Example of a “sparsely populated” subdomain, defined as a stack of outlines of sparse ipsilateral termini **(D)** in which outlines in neighboring sections overlap by at least 1%. **(C)** Example of a “diffuse subdomain,” in which the outlines overlap less than 1% in the anterior-posterior (*z*) axis, yet the entire group of outlines are close to one another relative to the distance to the closest neighboring subdomain. **(E)** Example of “stray outlines,” defined as outlines which neither overlapped with outlines in other sections, nor formed part of a group meeting criteria of a diffuse subdomain; these outlines were assigned to the closest subdomain in the dLGN, in this case, **(A)**.

The overlap criteria for grouping outlines depended on whether the termination zones were compact or sparse. When dLGN regions contained dense termini (overlapping or up to 10 μm apart, Figure 1C) we required that each consecutive stacked outline overlap with its neighboring section by at least 10% in area (Figure 2A). When an outlined region contained sparse termini (Figure 1D), its boundary was by nature ill-defined due to low sampling. For this reason, the distance to the closest terminal in the next section had to be large relative to the within section distance for the sections to be considered a separate subdomain. Therefore, while a sparsely populated subdomain still had to contain at least eight sections and be separated by approximately 75 μm from its closest neighbor, the alignment criteria was relaxed, and the outlines only had to overlap 1% with adjacent sections (Figure 2B).

In some cases, outlines aligned less than 1% with neighboring sections, or did not fall in groups of eight or more outlines. When a loose stack of these “stray outlines” was more closely grouped together than with any neighboring subdomain, these outlines were assigned to a “diffuse subdomain” (see Figure 2C). Otherwise, these stray outlines were incorporated into the closest neighboring subdomain (Figure 2D).

Finally, in a few cases, stray outlines displayed some properties of a distinct subdomain but did not meet all criteria. In this case, as illustrated in Figure 2E, these outlines were therefore grouped with closest subdomain (Figure 2A).

For three of the specimens (Rats 5–7), we also imaged and traced the retinorecipient ventral lateral geniculate (vLGN, including the Inter Geniculate Leaflet or IGL), and the retinorecipient layer of the optic tectum for volume comparisons.

### Calculation of volumes

To estimate the volumes of structures on the basis of traced outlines, we used the method of Sackett et al. (1989) as implemented by Najdzion et al. (2009). For the nth section, the sub-volume *V*_n_ is given by:

(1) $$V_{\text{n}}=\frac{\text{distance}\;\text{between}\;\text{sections}}{\text{3}}\times \left(a_{\text{n}}+a_{\text{n + 1}}+\sqrt{a_{\text{n}}}\times a_{\text{n + 1}}\right)$$

where *a*_n_ is the area of the cross section through the structure of interest based on the traced outlines. The sub-volumes of the extreme sections (end poles) were estimated as:

(2) $$V_{\text{n}}=\frac{\text{distance}\;\text{between}\;\text{sections}}{\text{3}}\times a_{\text{n}}$$

The total volume of the structure V_0_ is estimated by the sum of the sub-volumes throughout the region of interest:

(3) $$V_{0}=\sum V_{\text{n}}$$

This method was used to estimate the volume of the dLGN, the vLGN/IGL, and the SC (see Table 1). Due to the fragmented structure of the ipsilateral projection zones, we were not able to estimate the volume of the ipsilateral, and contralateral projection zones by this method. Instead we compared the areas of ipsilateral and contralateral projection zones over all traced sections.

**Table 1:** **Volumes of retinorecpient structures**.

Subject #	Left dLGN	Right dLGN	Left IGL/vLGN	Left optic tectum
1	1.76 mm^3^	1.56 mm^3^	–	–
2	1.63 mm^3^	1.59 mm^3^	–	–
3	1.55 mm^3^	1.59 mm^3^	–	–
4	1.66 mm^3^	1.63 mm^3^	–	–
5	1.53 mm^3^	–	0.55 mm^3^	3.70 mm^3^
6	1.46 mm^3^	–	0.69 mm^3^	3.71 mm^3^
7	1.42 mm^3^	–	0.65 mm^3^	3.51 mm^3^

*Volumes of retinorecipient structures were determined from traced outlines of retinal termination zones (see Methods). Subjects 1, 2, 3, and 4 were binocularly injected, and therefore the volume of the dorsal lateral geniculate nucleus (dLGN) could be measured on both sides of the brain. There was no significant difference in volume between right and left dLGN. Subjects 5, 6, and 7 were monocularly injected, so volumes could only be measured on the side contralateral to the injection. In these subjects, however, the entire brain was sectioned and imaged so that the optic tectum and the ventral lateral geniculate nucleus (vLGN)/intergeniculate leaflet (IGL) could also be reconstructed*.

### Image pre-processing for analysis of segregation

**Figure 3:**
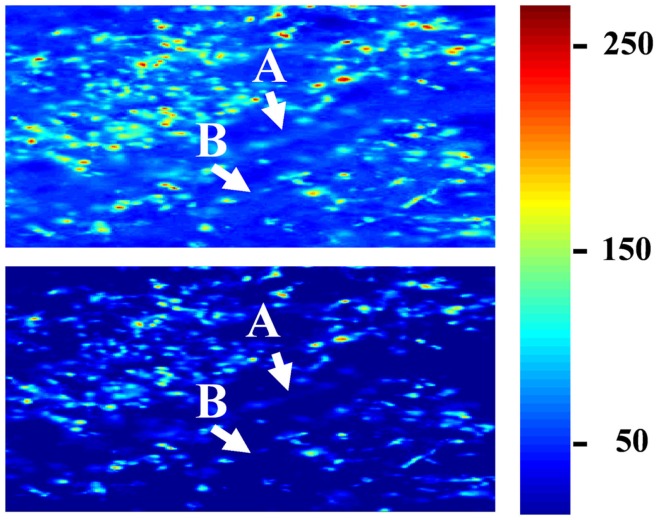
**Subtraction of background fluorescence from dLGN images**. Close up of an example image before background subtraction (top panel) and after background has been removed (lower panel). Pseudo-color indicates staining intensity, normalized to the range 0–255. Background threshold was chosen such that fibers of passage **(A,B)** were removed. This pre-processing step was used only for the segregation analysis (Figures 10–13), and ensured that only retinal termini contribute to calculations of binocularity.

To determine the extent to which termini from the two eyes segregate or overlap, the relative fluorescence was quantified at each location in the image. For this analysis images were preprocessed as follows. First, all fluorescent images throughout the brains were masked using the Neurolucida outlines in order to contain the dLGN only. Second, each image was corrected for bleed through fluorescence (crossover), which exists due to the overlap of the spectral profiles of the AlexaFlour 488 and 594 dyes. Specifically, the green fluorophor has some emission in the band captured by the red filter cube. In general this signal was negligible, but at the locations that were most intensely stained with the green fluorophor, the resulting signal in the red channel was significant relative to that of the comparatively weaker-staining red fluorophor. To remove the resulting artifact, we identified all pixels containing 95% of the maximum green staining, and set the red intensities at those locations to zero. Images were then visually inspected for successful artifact removal. Third, we thresholded the images to remove background fluorescence in order to exclude fibers of passage. The threshold for each section was set to 99 to 99.9 percentile of the intensity values taken from other regions of the same brain section known to contain RGC fibers of passage but not termini. Thresholded images were visually inspected to confirm that fibers of passage in the dLGN were removed while termini were spared (Figure 3). Based on this inspection, the percentile cutoff was manually adjusted for each brain as needed, but then the percentile was held constant for all sections from that brain. After background subtraction, each of the two fluorescent channels was normalized to the maximum intensity of that channel in all sections of that brain.

### Measure of segregation of inputs by eye of origin

To determine the overlap or segregation of ipsilateral and contralateral terminals, we used the analysis method of Torborg and Feller (2004), implemented in custom MATLAB code. For each pixel in the masked image we computed an *R*-value:

(4) $$R=\log\left(\frac{\text{intensity}\;\text{of}\;\text{ipsilateral}\;\text{staining}\;\text{of}\;\text{pixel}}{\text{intensity}\;\text{of}\;\text{contralateral}\;\text{staining}\;\text{of}\;\text{pixel}}\right)$$

Although *R* is continuous in value, for the purpose of summarizing results we classified an LGN location as “monocular” when staining from the non-dominant eye was <1% that of the dominant eye, corresponding to an *R*-value >2 or <−2. Thus any location with 1% or more contribution from the non-dominant eye (−2 ≤ *R* ≤ 2) was classified as binocular. This criterion is meant to be stringent with regard to our claim of strict segregation.

We observed uniform staining across the entire dLGN and optic tectum for Rats 1, 2, and 3, indicating complete filling of RGCs across the retina. In Rat 4, however, uneven and weak staining was observed in both retinal targets, indicating uneven filling of RGCs. It was still possible to visualize terminals clearly enough to manually outline the dLGN and termination zones by eye of origin, but this specimen did not pass the criterion for input segregation analysis, which depends on comparison of staining intensity.

### Control for low or unequal staining intensities

One possible confound to our analysis of segregation is that in many cases, staining intensities of one or more of the fluorescent CTBs was weak. While CTB is known for complete filling of RGCs (Angelucci et al., 1996; Reiner et al., 1996; Matteau et al., 2003), it is also known for its frequently low-intensity fluorescence as well as degradation over time (Angelucci et al., 1996). For the analysis of segregation we only included the three binocularly injected subjects in which staining was uniform throughout the major retino-recipient zones (see above). Nevertheless staining of the two fluorophores was generally unequal, with the red channel staining more weakly. Each channel was normalized to its own peak staining prior to the analysis of segregation (see above). Nevertheless, when staining is weak, it is possible that after subtracting background and fiber-of-passage fluorescence, some signal from the RGCs might have been missed. This could have biased the binocularity conclusions in favor of segregated eye inputs.

**Figure 4:**
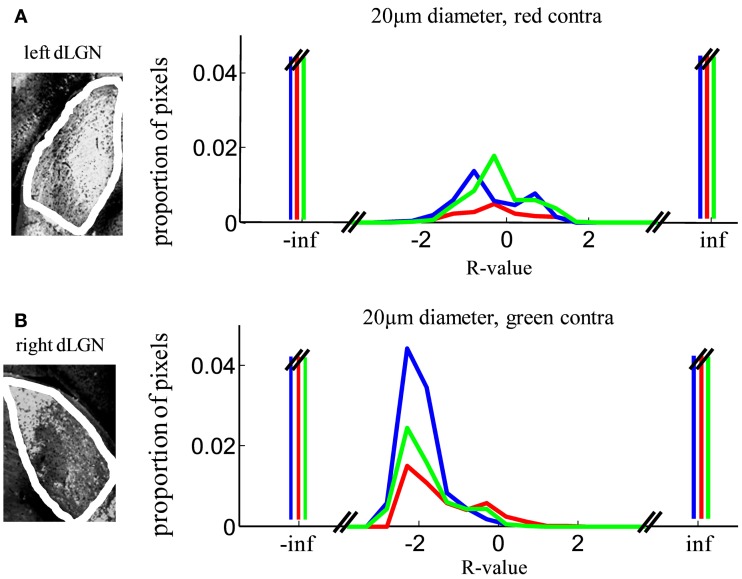
**Robustness of segregation analysis to unequal staining in the two eyes**. Eye of origin segregation was assessed by the log of the ratio of the intensity of ipsilateral to contralateral staining (*R*-value, see Methods). Here we show the same data as Figure 10, separated out based on hemisphere. **(A)** *R*-value distributions of left dLGN samples (three subjects), in which contralateral termini were stained red. **(B)** *R*-value distributions of right dLGN samples, in which contralateral termini were stained green.

Therefore as an additional control, we analyzed the *R*-value distributions for each dLGN separately. In one dLGN the weaker stain represents the contralateral projection while in the other dLGN weaker stain represents the ipsilateral projection. *R*-distributions with red-stained contralateral input (Figure 4A) showed far fewer pixels classified as contralateral monocular, compared with the dLGN with green-stained contralateral input (Figure 4B). Despite this asymmetry, both samples support our main conclusion that few locations in the dLGN have equal staining (*R* ∼ = 0), and a minority of locations have binocular staining (−2 < *R* < 2).

## Results

### Imaging retinal termini

We injected fluorescently conjugated CTB binocularly in four male Long–Evans rats in order to label retinal termini. Brains were later perfused and the region containing the dLGN sliced coronally into 25 μm thick sections and imaged using a Nanozoomer 2.0 HT (see Materials and Methods). The resulting images are wide field (multiple entire sections contained in a single scanned image) and high resolution (0.46 μm^2^/pixel). All inputs from the left eye fluoresced green (488 nm), and all inputs from the right eye fluoresced red (594 nm).

**Figure 5:**
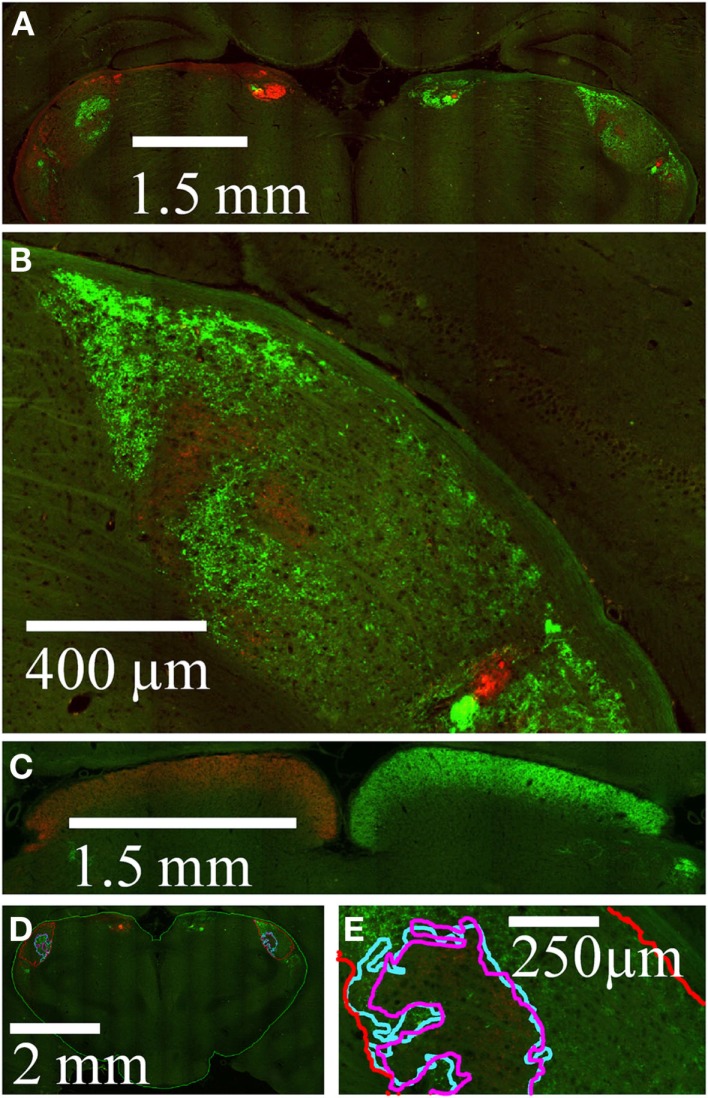
**Imaging of retinal termination zones**. High resolution wide field images of retinal termini in subcortical targets. AlexaFleur 488 nm-conjegated CTB (green) was injected in the left eye and AlexaFleur 594 nm-conjugated CTB (red) in the right eye. **(A)** A field of view within a coronal section from Rat 1, in which both dLGN as well as other retinal targets are visible. **(B)** Expanded view from the section in **(A)**, showing the right dLGN. **(C)** Field of view from a different coronal section from Rat 1, showing retinal projections to the optic tectum. **(D)** Field of view from a different section showing manually traced outlines of the entire subcortex (*green*), entire dLGN (*red*), holes in the projections from the contralateral eye (*magenta*), and projections from the ipsilateral eye (*cyan*). **(E)** Expanded view from **(D)** at the high resolution used to identify retinal termini during tracing, showing in the relationship between the contralateral hole and the ipsilateral projection outlines in this section.

A representative coronal section through the dLGN is shown in Figure 5. Viewed at moderate magnification, both left and right dLGN are visible, along with other retinorecipient structures in the subcortex (Figure 5A). The right dLGN is shown at higher magnification in Figure 5B. Stained retinal termini were also seen throughout other major retinorecipient targets, notably the optic tectum (Figure 5C).

Three additional rats were monocularly injected with non-conjugated CTB for analysis by light microscopy (not shown). Brains of these subjects were sliced coronally at 30 μm, termini stained with DAB, cell bodies counter-stained with Giemsa, and digitally imaged by light microscopy using the Aperio Scanscope (see Materials and Methods). The resulting images are wide field (multiple entire sections contained in a single scanned image) and high resolution (0.5 μm^2^/pixel). In these specimens we sectioned and imaged the entire brain, enabling volumetric analysis of additional retinal targets.

### Tracing retinal projections

Contralateral and ipsilateral retinal projection zones were traced in the dLGN on both sides of the brain (Figure 5D). Tracing was performed using the highest available magnification (Figure 5E) according to defined procedures and criteria (see Materials and Methods; Figure 1).

We traced every section through the LGN and surrounding brain for one binocularly injected, fluorescently labeled sample. We traced every fourth section through the LGN and surrounding brain for an additional three binocularly injected, fluorescently labeled samples, and three monocularly injected, non-fluorescent samples.

The resulting outlines were used to determine the volume of the dLGN and other structures (Table 1), to determine the three-dimensional structure of ipsilateral subdomains in the dLGN (Figures 6–9), and to determine the extent of overlap of the ipsilateral and contralateral projections to the dLGN (Figures 10–13).

**Figure 6:**
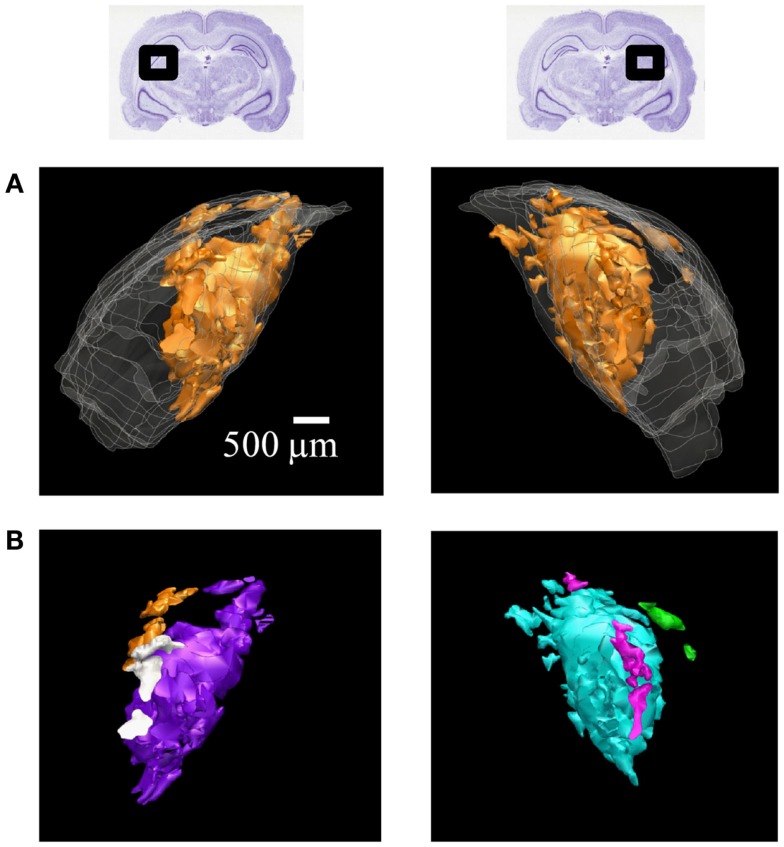
**3D reconstructions of ipsilateral subdomains within the dLGN of one rat**. A 3D reconstruction of the left (left) and right dLGN (right) of Rat 1, as schematically indicated by inset Nissl-stained sections above. For this subject only, every section through the brain was traced for higher resolution in the *z*-axis. **(A)** All the ipsilateral-recipient subdomain volumes shown in *orange*; the outline of the entire dLGN shown in translucent *white*. **(B)** The same ipsilateral subdomains shown in **(A)**, but each spatially separate subdomain is indicated in a different color. No specific correspondence between particular left and right subdomains is claimed, so different colors are used for the two hemispheres. A rotational view of this same 3D reconstruction is available in Movie S1 in Supplementary Material.

### Volume of the dLGN

In our samples the average volume of the dLGN was 1.58 mm^3^ ± 0.094 mm^3^ (mean ± SD, *n* = 11 dLGN nuclei from seven rats; Table 1). The dLGN comprised 70.0% (±3.0%, *n* = 3) of the total RGC-recipient geniculate volume, which includes the vLGN, the intergeniculate leaflet (IGL), and the dLGN. The volume of the dLGN was 40.4% (±1.0%, *n* = 3) that of the optic tectum.

### Putative ipsilateral subdomains within the dLGN

The area of the dLGN receiving ipsilateral input was 12.08 ± 1.82% (mean ± SD, *n* = 8) of the retinorecipient dLGN. In many sections, we observed two or more spatially separated zones of ipsilateral termini (e.g., Figure 5B), rather than the single compact termination zone expected based on the literature. These ipsilateral zones were well-aligned holes in the contralateral projections (Figure 5E). To determine whether these were part of a single connected three-dimensional (3D) ipsilateral-recipient zone, we reconstructed the dLGN and its retinal termination zones in 3D for all four binocularly injected subjects.

The contralateral and ipsilateral projection volumes of one subject are shown in Figure 6A. We found several spatially separated subdomains of ipsilateral termini within each dLGN (Figure 6B; Movie S1 in Supplementary Material), based on criteria that favored lumping over splitting (see Materials and Methods).

**Figure 7:**
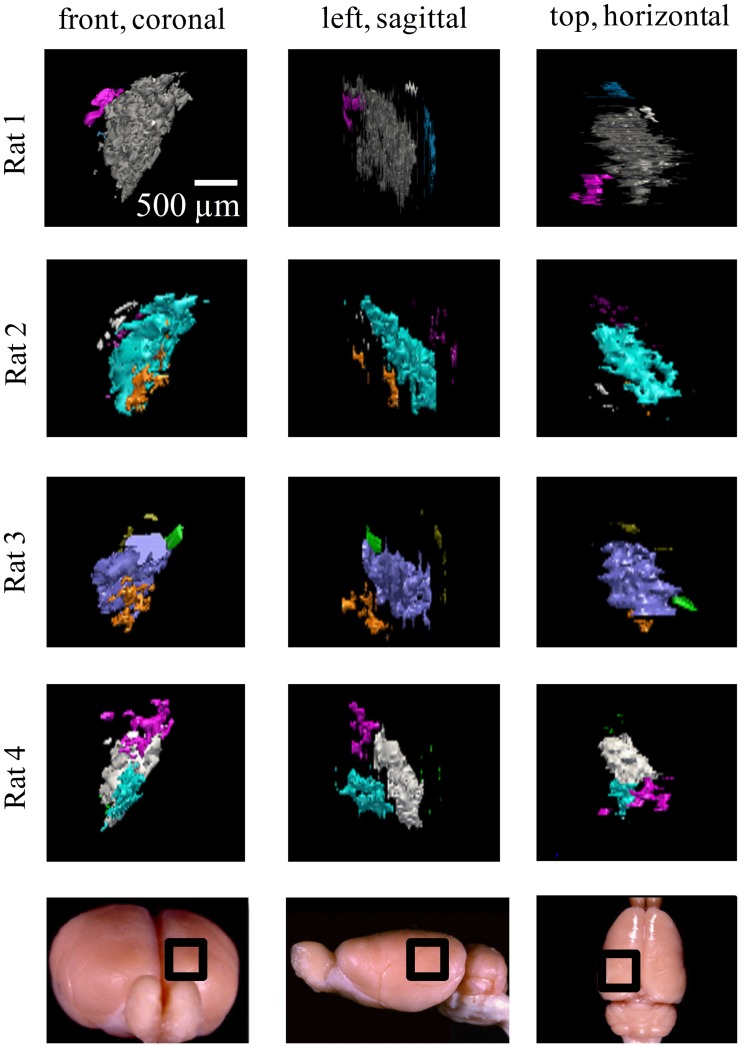
**Ipsilateral projections to the left dLGN of four subjects**. All the ipsilateral-recipient subdomain volumes in the left dLGN of each binoncularly injected subject, with each spatially separate ipsilateral subdomain indicated in a different color. Each 3D reconstruction is shown from three different vantage points: the top, front, and side view. The vantage point is illustrated at the bottom, with the dLGN position marked by a black square on a whole-brain icon. No specific correspondence between particular subdomains of different subjects, nor across hemispsheres, within subjects is claimed, so different colors are used for each subject and hemisphere.

**Figure 8:**
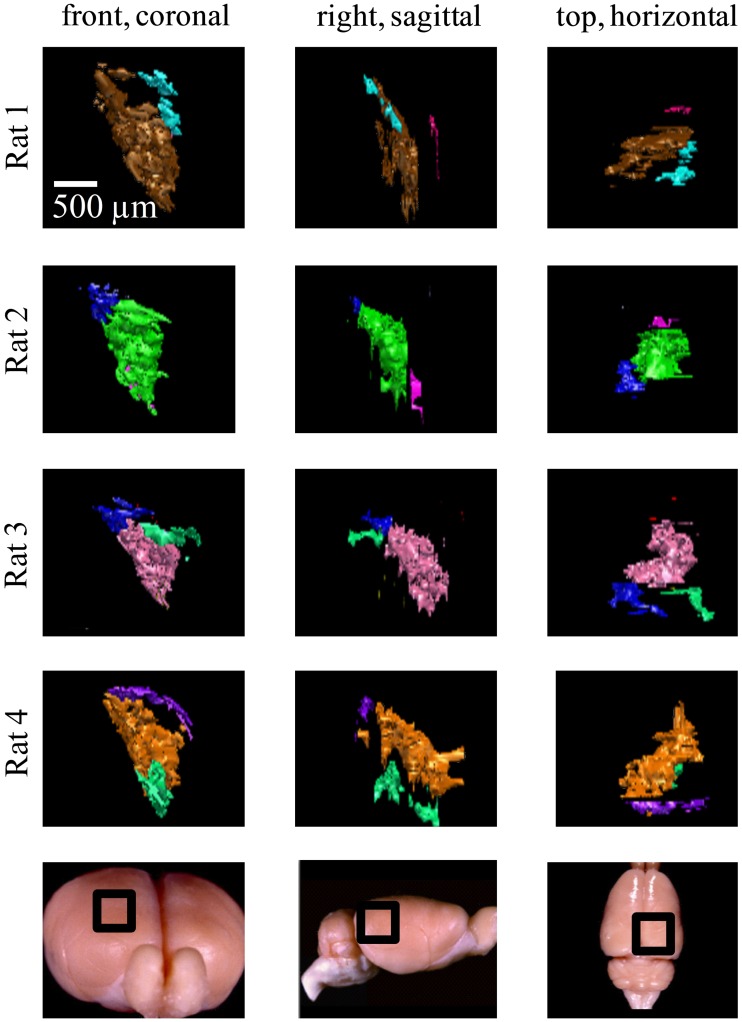
**Ipsilateral projections to the right dLGN of four subjects**. All the ipsilateral-recipient subdomain volumes in the right dLGN of each binoncularly injected subject, with each spatially separate ipsilateral subdomain indicated in a different color. Each 3D reconstruction is shown from three different vantage points: the top, front, and side view. The vantage point is illustrated at the bottom, with the dLGN position marked by a black square on a whole-brain icon. No specific correspondence between particular subdomains of different subjects, nor across hemispsheres, within subjects is claimed, so different colors are used for each subject and hemisphere.

Similar results were found in all four subjects (left hemisphere dLGN, Figure 7; right hemisphere dLGN, Figure 8). In general, three categories of ipsilateral subdomains were found: a dorsal-medial, a ventral-rostral, and a larger central region. We show the reconstructions for both hemispheres of all subjects from three perspectives, to allow direct inspection of the degree of bilateral symmetry as well as inter-subject variation.

**Figure 9:**
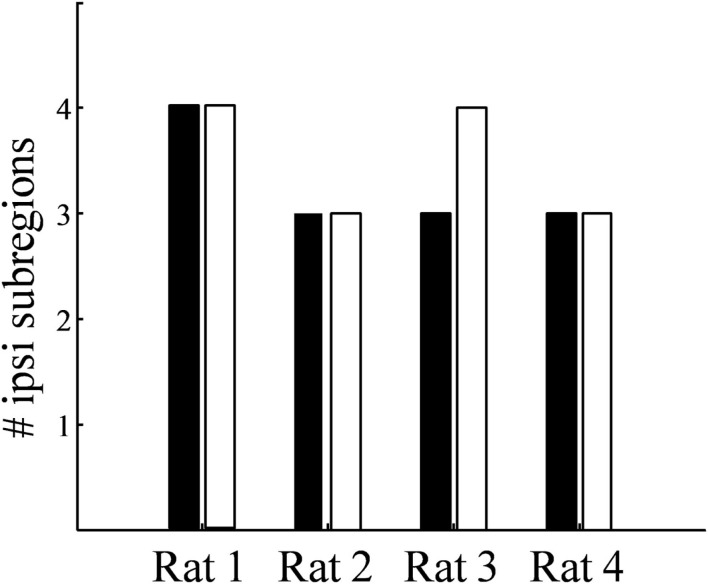
**Number of spatially separated ipsilateral-recipient subdomains in the dLGN**. For each binocularly injected subject (Rat 1–Rat 4), the number of identified subdomains in the left dLGN (*black*) and right dLGN (*white*) as determined by 3D reconstruction.

While the number of these subdomains (Figure 9) and their exact locations varied from animal to animal and even between hemispheres in the same animal (Figures 7 and 8), the approximate locations of these ipsilateral subdomains remained generally consistent. From these data we conclude that the dLGN of the pigmented rat typically contains multiple spatially separated ipsilateral projection zones, and not one single zone as described previously.

### Spatial segregation of retinal termination zones within the dLGN

We found little overlap in the traced outlines of ipsilateral and contralateral projection zones in the dLGN, consistent with strict segregation by eye of origin as described in other mammals. Considering that binocular responses have been reported in the literature (Grieve, 2005), however, the degree of segregation has been questioned. To address this further, we used a method introduced by Torborg and Feller (2004) to measure segregation using the relative intensity of staining of retinal termini originating from the two eyes. We computed for each location in the dLGN an index of binocularity *R*, defined as the log of the ratio of ipsilateral to contralateral staining (see Materials and Methods, Eq. 4). The index *R* has a negative value when contralateral inputs are stronger, a positive value when ipsilateral inputs are stronger, and is 0 when the normalized intensity originating from the two are equal.

**Figure 10:**
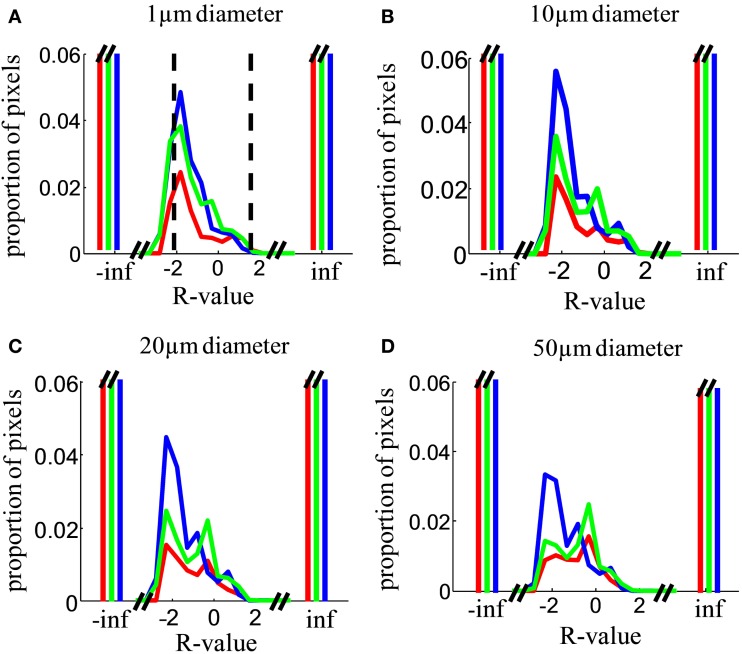
**Relative contribution of inputs from the two eyes**. **(A)** Distribution over locations in the image of the *R*-value, i.e., the log of the ratio of ipsilateral to contralateral staining intensity (Eq. 4), analyzed in a sampling diameter of 1 μm. Distributions were computed over the entire area of the dLGN in both hemispheres in all analyzed sections. Results are shown for Rat 1 (*green*), Rat 2 (*red*), and Rat 3 (*blue*). The cutoff values for our operational definition of “binocular” (−2 < *R *< 2) are indicated by vertical *dashed lines*. Distributions were normalized such that the histograms sum to 1. The *y*-axis is greatly expanded to show the shape of the distribution for the small minority of pixels with detectable staining from both eyes; the proportion of monocular pixels (*R* = ∞ or *R* = −∞) is far off-scale. **(B–D)** As in **(A)**, but analyzed in a sampling diameter of 5, 10, and 20 μm respectively.

**Figure 11:**
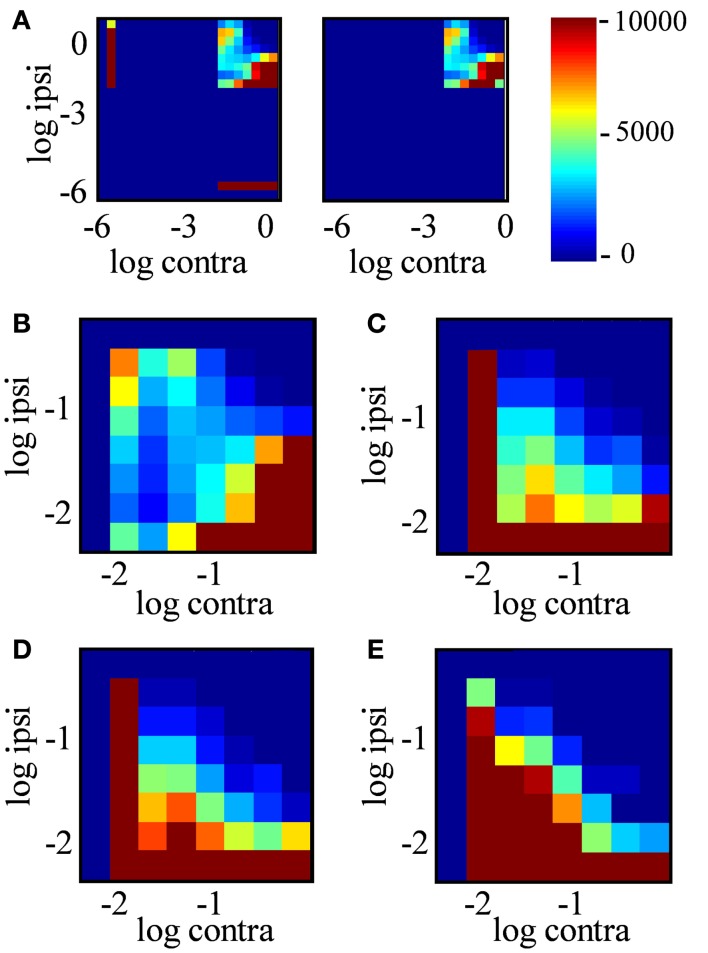
**Joint distribution of ipsilateral and contralateral input strength**. **(A)** Joint probability distribution of log contra staining intensity (*x*-axis) vs. log ipsi staining intensity (*y*-axis) over positions in the dLGN, where color indicates the number of locations in the dLGN with these staining intensities. Data are from the entire dLGN, both hemispheres, all sections of Rat 1, using a sampling diameter 20 μm. Intensity was discretized in bins of 0.3 log units^2^. The *left subpanel* includes all positions in dLGN; the *right subpanel* includes only “binocular” locations (−2 < *R *< 2). **(B–E)** Expanded view of the joint probability distribution for binocular locations only, analyzed in a spatial sampling diameter of 1, 5, 10, and 20 μm respectively.

**Figure 12:**
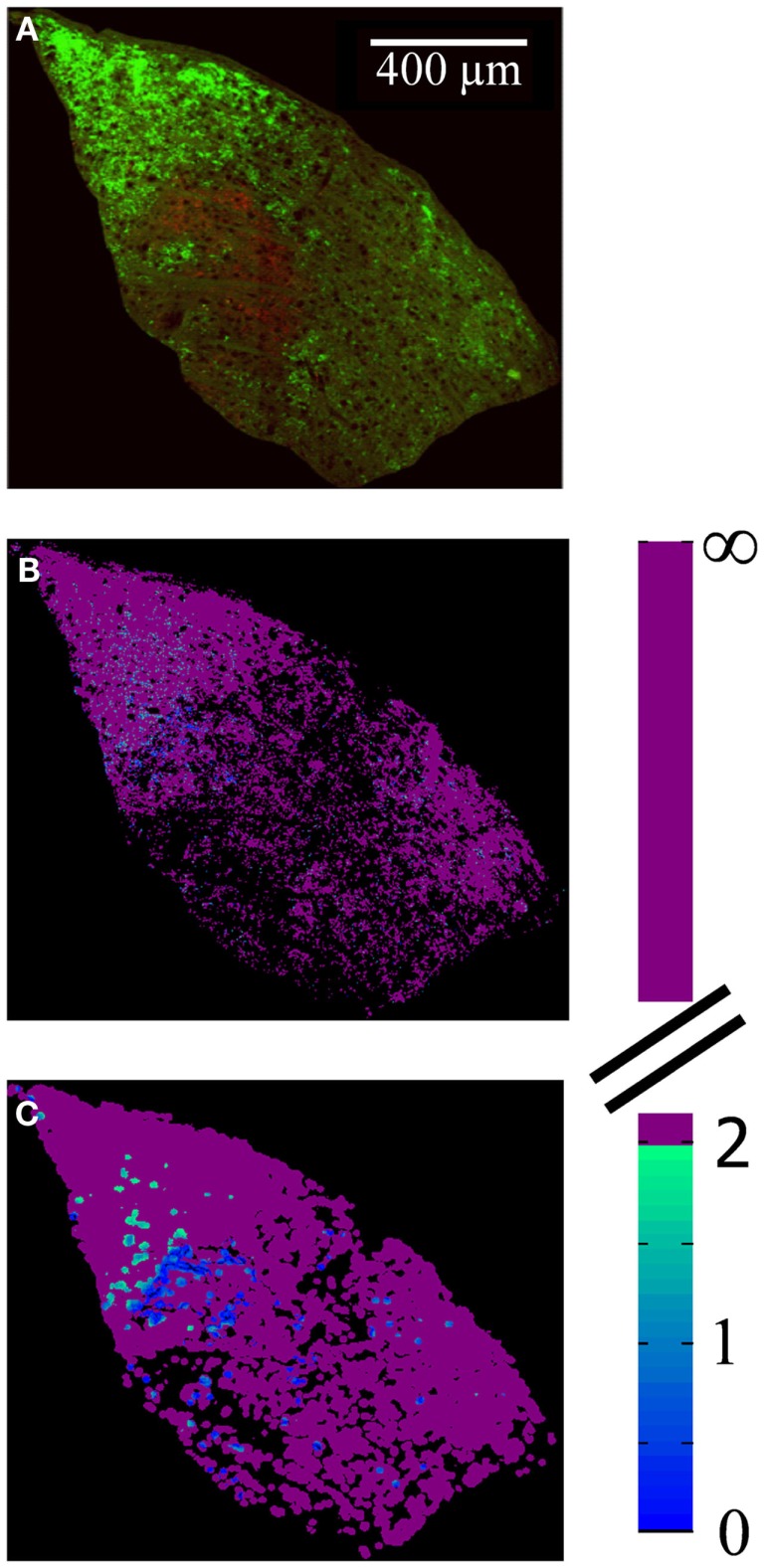
**Spatial distribution of locations with input from both eyes**. **(A)** Merged image of the contralateral (green) and ipsilateral (red) retinal termini, as labeled by the fluorescence of conjugated CTB tracers, in a representative slice through the dLGN. Brightness and contrast have been adjusted for illustration purposes; area outside the dLGN has been masked out. **(B,C)** False-colored images of same section, where color indicates the absolute value of *R* (color scale at right), for a spatial sampling diameter of 1 μm **(B)** and 20 μm **(C)**.

**Figure 13:**
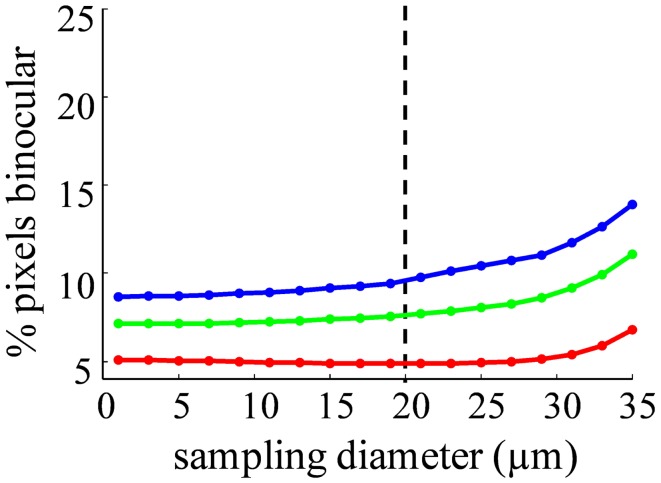
**Probability of an area containing terminals from both eyes depends on sampling diameter**. The percentage of binocular pixels (−2 < *R *< 2) as a function of the spatial sampling diameter, for Rat 1 (*green*), Rat 2 (*red*), and Rat 3 (*blue*). Our estimate of the diameter of a rat dLGN cell soma is shown in the *dashed line*.

The result of this analysis will depend critically on the spatial sampling diameter over which intensity is measured. In the limit of analyzing single submicron pixels, each “location” is smaller than a single retinal terminal, so contributions at that spatial scale will be monocular, even in a binocular structure with completely mixed, unsegregated inputs. In the limit of large sampling diameter, a single “location” could include the entire dLGN, and contributions will be binocular even for a structure with well segregated inputs. In general, we expect binocularity to increase with sampling diameter. The choice of sampling diameter is somewhat arbitrary, so we present results as a function of this variable (Figures 10–13). Our findings are robust to choice of this parameter.

At a sampling diameter of 1 μm, over 90% of locations in the dLGN have monocular input (no measurable staining originating from the other eye). Some locations, however, have measurable staining in both channels, implying at least some retinal termini from each eye of origin (Figure 10A). We operationally defined locations with −2 < *R* < 2 as “binocular”; locations with *R* ≤ −2 as monocular and contralateral; and locations with *R* ≥ 2 or greater as monocular and ipsilateral. This classification is meant to be stringent relative to a claim of segregation: if even 1% of the staining originates from the non-dominant eye the location is considered binocular, even though no relay cell may in fact sample from termini of both eyes at that location.

Across a wide range of sampling diameters (1–50 μm, Figures 10A–D), most of the dLGN locations classified as binocular have stronger input from the contralateral eye (peak near *R* = −2, corresponding to 100:1 excess of contralateral staining). The contralateral contribution was stronger (peaked at *R* < 0) regardless of whether the contralateral eye was the weaker or the stronger staining (see Materials and Methods, Figure 4). Additional smaller peaks were often observed near *R *= 0 (equal contribution) and near *R *= 0.5–1 (ipsilateral dominating by 3- to 10-fold).

In principle, an *R-*value near 0 (staining ratio near 1) could arise from extremely weakly stained locations in both channels; the ratios of very small numbers would not be reliable due to noise. The joint histogram of staining intensities in the two eyes (Figure 11) reveals, however, that most locations classified as binocular arose from locations with clearly measurable staining in both eyes (log intensities > 2 in both channels, corresponding to ≥1% of maximum intensity in each channel). Most dLGN locations classified as monocular by our criteria had no detectable staining (intensities <10^−6^) in the non-dominant eye (Figure 11A, compare left vs. right panels). As the sampling diameter increased from 1 to 20 μm (Figures 11B–E), so does the number of locations in the dLGN that show equal contributions from the two eyes (density along *x* = *y* diagonal). Yet up to a sampling diameter of 20 μm, most binocular pixels were dominated by either contralateral or ipsilateral input (off-diagonal density).

The spatial distribution of *R*-value reveals that most dLGN positions classified as binocular lie at the boundaries between monocular regions (Figures 12A–C). At a sampling diameter of 20 μm, for example, only 5% of pixels in the section shown were classified as binocular, and most of these fell along the boundaries between ipsilateral- and contralateral-recipient regions (Figure 12C). The percentage of pixels classified as binocular is shown as a function of sampling diameter for all three subjects (Figure 13). At a sampling diameter of 20 μm, between 90–96% of all positions were classified as strictly monocular.

## Discussion

The data and analysis presented here confirm the basic findings of an earlier preliminary report (Discenza et al., 2008): retinal projections to the dLGN of the rat are well segregated by eye of origin, and the ipsilateral projections form multiple spatially separated subdomains.

### Volume of dLGN

The volume of the dLGN relative to the entire retinorecipient thalamus (dLGN, IGL, and vLGN) has been related to the visual sophistication of species. Najdzion et al. (2009) found that the contribution of the dLGN to the total LGN volume was 57% in the common shrew and 50% in the bank vole, which are both nocturnal and partially subterranean species. The relative size of the dLGN was considerably larger in the more visually dependent rabbit (64%) and fox (95%). Here we found the rat dLGN was 70 ± 3% of the LGN, placing it closer to the highly visual end of the spectrum of mammals (Table 1).

Brauer et al. (1982) found that among 16 species, within a given order, those with a high level of neocorticalization also tended to have a high ratio of dLGN to vLGN volumes. In addition, ratios of dLGN to vLGN size were positively correlated with extent of dLGN lamination. The high ratio of dLGN to vLGN we found in the rat would be consistent with a laminated dLGN, despite the absence of obvious structural laminae. The entire retinorecipient thalamus was nevertheless smaller than the volume of the retinorecipient tectum (58 ± 1%, *n* = 3).

### Multiple ipsilateral termination zones

Ipsilateral projection zones comprised 12% of the area of the retinorecipient dLGN, consistent with the proportion of RGC crossover in the optic chiasm, as well as the percentage of binocular overlap in the rat’s field of vision.

Rather than a single ipsilateral domain, three or four spatially separated subdomains of ipsilateral input were consistently found in each dLGN (Figure 9). These subdomains were typically seen dorsal-medially, centrally, and rostral-ventrally, though the exact locations and volumes were not well-conserved between subjects or even across hemispheres of the same brain. The presence of spatially separated ipsilateral subdomains raises the possibility of multiple interleaved ipsilateral and contralateral sublaminae.

In other mammals, sublaminae that represent parallel processing streams in the dLGN are sometimes but not always distinguishable in Nissl-stained sections on the basis of soma size and density. The putative subdomains we identified on the basis of termination zones appeared similar in these characteristics under Nissl stain; a quantitative analysis described elsewhere failed to find any statistical difference in soma size or density between different identified subdomains (Discenza, 2011). However, this does not preclude the existence of morphologically, functionally, or architecturally distinct subdomains, which may yet be revealed by other methods. In particular, we have not determined the retinal ganglion cell terminal morphology, which reveals hidden sublamination of the dLGN of other species (Major et al., 2003).

Functionally distinct sublaminae are expected to receive input from distinct RGC subtypes. Several early studies found general differences in the anatomical types of RGCs that project to the “outer shell” vs. the “inner core” of the dLGN (Martin, 1986; see Reese, 1988). These studies found that type I (alpha) RGCs (cells with large somas and 3–6 primary branching dendrites) synapsed in the dLGN “inner core,” type II (B) cells (small somas with short dendrites) synapsed throughout the nucleus, and type III (C) RGCs (cells with smaller somas and very long dendrites) were found in the “outer shell” only. But there are also at least a dozen functional subclasses of RGCs in the rat, each transmitting their own distinct information (Yonehara et al., 2009). Using new techniques to trace individual functional cell types, such as molecular tags (Marc and Jones, 2002) and genetic markers (Huberman et al., 2008, 2009), one could test whether the spatially separated ipsilateral domains receive projections from distinct RGC populations.

If the ipsilateral subdomains we describe here represent distinct functional laminae, they should contain separate retinotopic maps. Alternatively, if they represent a single spatially fragmented layer, they should jointly contain a single retinotopic map. The retinotopy of the rat dLGN has been described from physiological data (Reese and Cowey, 1983; Reese and Jeffrey, 1983; Reese, 1988). But given the small size and variable position of the ipsilateral subdomains, it would be necessary to fill and reconstruct the recorded cells to make any detailed comparison of retinotopy or physiological properties between subdomains. Retrograde labeling from V1 would provide valuable information about the retinotopic map(s) in the ipsilateral subdomains.

### Segregation of inputs by eye of origin

Early studies reported that ipsilateral and contralateral retinal projections are segregated in the mature rat dLGN (Reese and Cowey, 1983; Reese and Jeffrey, 1983; Reese, 1988). One recent study, however, reported that up to 63% of relay cells in the rat dLGN respond to direct stimuli from either eye (Grieve, 2005), casting some doubt as to the degree of segregation of inputs from the two eyes.

While it is known that dLGN relay cell dendrites span nearly the entire nucleus (Gabbott et al., 1986) it has been shown that relay cells only synapse with RGC termini close to the soma (Hamos et al., 1985; for review, see Sherman and Guillery, 1996). We previously estimated the average cell diameter in the rat dLGN to be 20 μm (Discenza, 2011); others reported a maximum diameter of 15 μm (Villena et al., 1997). If a dLGN relay cell samples retinal termini over a diameter of 20 μm, only 5–10% of locations within the dLGN have access to termini from both eyes, and most of these locations fell along the borders between ipsilateral- and contralateral-recipient regions (Figures 12 and 13). LGN relay cells receive inputs from only 1–5 RGC (Levick et al., 1972); therefore even if they sampled uniformly within this radius, most would still be monocularly innervated. If binocular responses of dLGN relay cells are confirmed in the rat, they would more likely be explained by non-retinal inputs.

### Rat as a model system for vision

Until relatively recently, rats have been regarded as largely non-visual animals, better known for their ability to use sense of smell and whisker-touch to navigate their environments (Hill and Best, 1981; Hutson and Masterton, 1986; Carvell and Simons, 1990; Maaswinkel and Whishaw, 1999; Save et al., 2000; Kulvicius et al., 2008). Yet despite their poor acuity and limited color vision (Jacobs et al., 2001; Prusky et al., 2002, for review see Burn, 2008), pigmented rats can learn and perform a wide range of visual tasks. In the laboratory setting, rats have demonstrated visuo-spatial learning and memory (Zoladek and Roberts, 1978; Morris, 1984), navigation (Holscher et al., 2005), and visual object detection and pattern discrimination (Thompson and Solomon, 1954; Zoccolan et al., 2009; Clark et al., 2011; Meier and Reinagel, 2011; Meier et al., 2011), as well as visually mediated fear conditioning (Shi and Davis, 2001) and eye-reflexes and movements such as nystagmus and saccades (Fuller, 1985; Hess et al., 1985; Hikosaka and Sakamoto, 1987).

The rat and mouse are increasingly important model systems for visual behavior and physiology, it will be important to understand more about the functional organization and connectivity of the early visual pathways in these nocturnal rodents.

## Conclusion

Our data reveal more anatomical organization in the rat dLGN than previously described. We confirm that inputs from the two eyes are well segregated in the rat dLGN. We find 3–4 geographically distinct ipsilateral subdomains in the largely contralateral dLGN. It remains to be determined whether these putative subdomains receive input from distinct classes of RGC or contain duplicate maps of retinotopic space.

## Conflict of Interest Statement

The authors declare that the research was conducted in the absence of any commercial or financial relationships that could be construed as a potential conflict of interest.

## Supplementary Material

The Supplementary Material for this article can be found online at http://www.frontiersin.org/Neuroanatomy/10.3389/fnana.2012.00040/abstract

Supplementary Movie S1 **Discrete Ipsilateral Projection Subdomains in the rat dLGN**. Rotational view of the 3D reconstructions of ipsilateral subdomains within the left dLGN of Rat 1 (Figures 6 and 7), shown rotating about the rostral-caudal axis of the dLGN.
